# Suppression of TRPV5 Regulates Microglia‐Mediated Neuroinflammation Following Status Epilepticus

**DOI:** 10.1002/glia.70068

**Published:** 2025-07-24

**Authors:** Soojin Park, Se Hoon Kim, Chul Hoon Kim, Kyoung Hoon Jeong, Won‐Joo Kim

**Affiliations:** ^1^ Department of Neurology, Graduate School of Medical Science, Brain Korea 21 Project Yonsei University College of Medicine Seoul Republic of Korea; ^2^ Department of Pathology Yonsei University College of Medicine, Severance Hospital Seoul Republic of Korea; ^3^ Department of Pharmacology, Brain Korea 21 Project, Brain Research Institute Yonsei University College of Medicine Seoul Republic of Korea; ^4^ Epilepsy Research Institute Yonsei University College of Medicine Seoul Republic of Korea; ^5^ Department of Neurology, Gangnam Severance Hospital Yonsei University College of Medicine Seoul Republic of Korea

**Keywords:** econazole, microglia, neuroinflammation, status epilepticus, TRPV5

## Abstract

Neuroinflammation, predominantly associated with glial activation and the release of various inflammatory mediators, is a vital hallmark of the pathophysiology of epilepsy. Numerous studies have indicated that identifying novel factors that diminish neuroinflammatory processes may be important for developing effective therapeutic strategies to prevent neuropathological processes and epileptogenic progression. Transient receptor potential vanilloid 5 (TRPV5) is a highly selective calcium ion channel belonging to the TRPV family. TRPV5 expression has been identified in diverse regions of the brain; however, it remains unknown how TRPV5 is implicated in the pathophysiological features of neurological diseases, including epilepsy. Herein, we show that TRPV5 expression is upregulated in the hippocampus of a pilocarpine‐induced status epilepticus (PCSE) model, predominantly in activated microglia. Pharmacological inhibition of TRPV5 using econazole attenuated microglial activation, as indicated by the shift of LPS‐stimulated primary hippocampal microglia to a resting state. This inhibition suppressed AKT/NF‐κB signaling, reduced NLRP3 inflammasome activity, and decreased proinflammatory cytokine production. Additionally, TRPV5 inhibition reduced hippocampal microglial activation and neuroinflammation following PCSE. These findings suggest that TRPV5 contributes to the regulation of microglial activation, resulting in the suppression of microglia‐derived neuroinflammation during the sub‐acute phase of epilepsy. In conclusion, the present study suggests that targeting TRPV5 may offer a novel therapeutic approach to managing the neuroinflammatory processes during epileptogenic progression.

## Main Points

- TRPV5 is involved in microglial activation following epileptic seizures.
- TRPV5 upregulation in microglia drives neuroinflammation.
- TRPV5 inhibition by econazole reduces neuroinflammation via suppressing AKT/NF‐κB/NLRP3 signaling cascade.

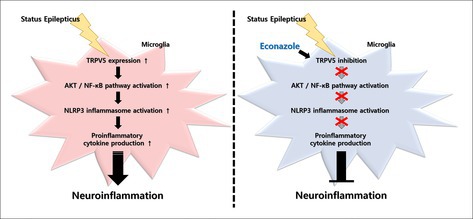

## 1 Introduction

Neuroinflammation is characterized by the activation of glial cells, including microglia and astrocytes, as well as peripheral immune cells, which release inflammatory mediators, such as cytokines, chemokines, and reactive oxygen species (DiSabato et al. 2016; Sochocka et al. 2017). While neuroinflammation functions as a defense mechanism to protect the brain by eliminating or suppressing various pathogens, uncontrolled inflammatory actions exacerbate neuronal injury, disrupt homeostasis, and trigger blood–brain barrier dysfunction, resulting in neuropathological progression (Kwon and Koh 2020; Muller et al. 2025; Takata et al. 2021; Ward et al. 2022). Therefore, excessive neuroinflammation has been implicated as a critical process in the pathophysiology of numerous neurological diseases, including neurodegenerative diseases, stroke, traumatic brain injury, and epilepsy (Mracsko and Veltkamp 2014; Ransohoff 2016; Simon et al. 2017; Vezzani et al. 2011). This suggests that managing neuroinflammatory processes could be essential for developing potent therapeutic strategies to prevent or treat central nervous system (CNS) diseases.

Epilepsy is a chronic neurological disorder characterized by recurrent spontaneous seizures that affect approximately 70 million individuals worldwide (Thijs et al. 2019). Neuroinflammation plays a pivotal role in numerous pathophysiological conditions related to the development of epilepsy, and it is both a cause and consequence of epileptogenesis (Rana and Musto 2018). This inflammatory process involves a complex interplay among neurons, microglia, and astrocytes, amplifying the pathological response (Sanz et al. 2024). In particular, microglia, the resident immune cells of the CNS, are pivotal mediators of the inflammatory cascade in epilepsy. Activated microglia release proinflammatory cytokines and chemokines, resulting in neuronal hyperexcitability, synaptic reorganization, and neuronal death (Vezzani and Viviani 2015). Elevated levels of inflammatory mediators have been observed in glial‐like cells in both animal models and resected brain tissue from patients with temporal lobe epilepsy (TLE) (Kan et al. 2012; Ravizza et al. 2008), indicating the pathological significance of microglial activation in driving neuroinflammation in epileptic brain damage. Accordingly, identifying and understanding novel molecular factors regulating microglia‐mediated neuroinflammation may be crucial for the development of effective therapeutic approaches for epilepsy.

The vanilloid subfamily of transient receptor potential (TRPV) channels has recently emerged as a promising therapeutic target for several neurological disorders (Lee et al. 2021). The TRPV subfamily further branches into TRPV1–6; among these, TRPV1 and TRPV4 have been implicated in the modulation of neuroinflammation and epileptogenesis. For example, TRPV1 activation enhances microglial activation and inflammatory responses, indirectly increasing seizure susceptibility in hyperthermia‐induced febrile seizures (Kong et al. 2019). Conversely, inhibition of TRPV4 reduces microglial and astrocyte activation, attenuates inflammatory responses, and decreases neuronal cell death in a pilocarpine‐induced status epilepticus (PCSE) model (Wang et al. 2019). These findings indicate that TRPV channels may play a critical role in reactive gliosis and inflammation triggered by epileptic activity. Within the vanilloid subfamily, TRPV5 is functionally distinct due to its highly selective permeability to calcium ions. Under physiological conditions, TRPV5 regulates calcium absorption and reabsorption in epithelial tissues, such as the kidney (van der Wijst et al. 2019). A previous study reported the involvement of TRPV5 in inflammatory regulation under pathological conditions like osteoarthritis. For instance, blocking TRPV5 reduces inflammatory cytokine expression in osteoarthritis patient‐derived chondrocytes (Zhong et al. 2021), indicating a potential role for TRPV5 in modulating inflammatory responses under pathological conditions. Interestingly, Kumar et al. reported the expression of TRPV5 in diverse regions of the rodent brain, particularly in the hippocampal CA1 and CA3 regions (Kumar et al. 2017). However, evidence linking TRPV5 to CNS disease‐related pathophysiological features remains limited.

Given the involvement of TRPV channels in reactive gliosis and neuroinflammatory processes following epileptic seizures (Kong et al. 2019; Wang et al. 2019), we hypothesized that TRPV5 might also be associated with glial activation and neuroinflammatory responses following epileptic injury. Therefore, in the present study, we investigated whether TRPV5 drives microglial activation during prolonged seizure activity and how TRPV5 participates in inflammatory processes during microglial activation, highlighting the potential role of TRPV5 as a therapeutic target for mitigating neuroinflammation.

## 2 Methods

### 2.1 Mouse Model of Pilocarpine‐Induced Status Epilepticus

All animal experiments were approved by the Institutional Animal Care and Use Committee (IACUC) at Yonsei University Health System (no. IACUC 2023–0208), and were conducted in accordance with the National Institutes of Health guidelines for the Care and Use of Laboratory Animals. Efforts were made to minimize animal suffering and reduce the number of animals used.

Male C57BL/6 mice (24–25 g; Orientbio, Gyeonggi, Korea) were housed under standard temperature and humidity conditions, under a 12 h light/dark cycle, with ad libitum access to food and water. A mouse model of PCSE was established, as previously described (Park et al. 2024). Following the acclimation period, the mice were injected with scopolamine methyl nitrate (1 mg/kg, i.p.; Sigma‐Aldrich, St. Louis, MO, USA) to minimize the peripheral side effects of pilocarpine treatment. After 30 min, pilocarpine hydrochloride (325 mg/kg, i.p.; Sigma‐Aldrich) was injected intraperitoneally to induce status epilepticus (SE). Sham‐manipulated animals were subsequently injected with an equivalent volume of saline as the controls. Behavioral seizures were assessed using the Racine scale (Racine 1972), summarized as follows: stage 1, facial movements; stage 2, head nodding; stage 3, forelimb clonus; stage 4, rearing; and stage 5, rearing and falling. Mice reaching stages 3–5 and showing continuous behavioral seizure with generalized tonic–clonic seizures were considered to have entered SE and were included in the experiment. All pilocarpine‐injected mice developed SE and reached Racine stage 3 or above. The mortality rate following pilocarpine‐induced SE was approximately 30%. To terminate seizure activity, diazepam (10 mg/kg; Samjin Pharm, Seoul, Korea) was administered 2 h following SE induction. Mice were placed in cages with bedding on a heating pad set to 37°C for faster recovery.

### 2.2 Brain Tissue Preparation and Immunofluorescence Staining

Following SE induction, the mice were euthanized under deep anesthesia with 40% urethane and then transcardially perfused with saline, followed by 4% paraformaldehyde (PFA). The brains were then isolated, post‐fixed in PFA overnight, and maintained in 30% sucrose solutions at 4°C until sedimentation. The brains were subsequently embedded in optimal cutting temperature compound (Tissue‐Tek, Sakura Finetek, Torrance, CA, USA) and frozen. Coronal brain sections were then cut at a thickness of 30 μm, collected between bregma −1.46 and −2.30 mm using a cryomicrotome (Leica Microsystems, Wetzlar, Germany), and collected in 0.01 M phosphate‐buffered solution (PBS).

Immunofluorescence staining was performed as previously described (Park et al. 2024). After blocking with 5% normal horse serum (Vector Laboratories, Burlingame, CA, USA) in 0.01 M PBS for 1 h at room temperature, tissue sections were incubated overnight at 4°C with a mixture of primary antibodies, including rabbit anti‐TRPV5, anti‐glial fibrillary acidic protein (GFAP), and anti‐ionized calcium‐binding adapter molecule 1 (Iba1) (Table 1). The following day, sections were incubated with fluorescence‐conjugated secondary antibodies (Table 1) for 2 h. The stained sections were then covered by a glass coverslip using a hard‐set antifade mounting medium containing DAPI (Vectashield; Vector Laboratories). The labeled sections were subsequently mounted on slides and observed under a fluorescence microscope (Axio Imager M2; Carl Zeiss, Thornwood, NY, USA). Images were captured using a confocal laser‐scanning microscope (LSM 700; Carl Zeiss).

**TABLE 1:** Details of the Primary and Secondary antibodies used.

Primary antibody	Dilution	Source
Rabbit anti‐transient receptor potential Vanilloid 5 (TRPV5)	1: 300 for IHC	Abcam; ab137028
	1: 2000 for WB	
Mouse anti‐glial fibrillary acidic protein (GFAP)	1: 300 for IHC	Millipore; MAB360
Goat anti‐ionized calcium‐binding adapter molecule 1 (Iba1)	1: 300 for IHC	Abcam; ab5076
Rabbit anti phospho‐protein kinase B (Ser473) (p‐Akt)	1: 1000 for WB	Cell signaling Technology; #9271
Rabbit anti‐AKT	1: 1000 for WB	Cell signaling Technology; #9272
Rabbit anti‐phospho‐nuclear factor‐κB (p‐NF‐κB)	1: 1000 for WB	Cell Signaling Technology; #3033
Mouse anti‐NF‐κB	1: 1000 for WB	Cell Signaling Technology; #6956
Mouse anti‐nucleotide‐binding and oligomerization domain‐like receptor family pyrin domain‐containing 3 (NLRP3)	1: 1000 for WB	AdipoGen; AG‐20B‐0014
Mouse anti‐apoptosis‐associated speck‐like protein containing a caspase‐recruitment domain (ASC)	1: 1000 for WB	AdipoGen; AG‐25B‐0006
Rabbit anti‐caspase1	1: 1000 for WB	AdipoGen; AG‐20B‐0042
Rabbit anti‐Interleukin‐18 (IL‐18)	1: 1000 for WB	Invitrogen; #PA5‐79481
Mouse anti‐β‐Actin	1: 4000 for WB	Santa Cruz Biotechnology; sc‐47,778

Secondary antibody	Dilution	Source
Cy3 AffiniPure Rabbit Anti‐Goat IgG (H + L)	1: 600	Jackson Immuno Research; 301–165‐003
Goat anti‐rabbit IgG antibody (H + L), DyLight 594	1: 600	Vector lab; DI‐1594‐1.5
Alexa Fluor 488 goat anti‐mouse IgG (H + L)	1: 600	Invitrogen; 2,659,299
Goat anti‐rabbit IgG, polyclonal antibody (HRP conjugate)	1: 10000	Enzo; ADI‐SAB‐300
Goat anti‐mouse IgG, polyclonal antibody (HRP conjugate)	1: 10000	Enzo; BML‐SA204‐0100
Mouse anti‐sheep/goat IgG monoclonal antibody (GT‐34) (HRP conjugate)	1: 10000	Enzo; ADI‐SAB‐400

### 2.3 Identification of Cellular Phenotype in TRPV5‐Expressing Cells

To identify the cellular phenotype of TRPV5‐expressing cells in the hippocampus following SE induction, Pearson's correlation coefficient analysis was performed by evaluating the degree of colocalization between TRPV5 and GFAP, as well as TRPV5 and Iba1, as previously described (Kong et al. 2019; Marrone et al. 2017). The correlation coefficients were interpreted as follows: < 0, negative; 0, none; 0–0.19, very weak; 0.20–0.39, weak; 0.40–0.59, moderate; 0.60–0.79, strong; and 0.80–1.0, very strong (Liu et al. 2024). For quantitative analysis, one hippocampal section was analyzed per animal, and a single region of interest was selected from the subpyramidal region of the CA1 area, where TRPV5 expression was most prominent. The analysis was performed using tissue from four independent SE animals.

### 2.4 Human Subjects and Immunofluorescence Staining

Human tissue experiments were conducted with informed consent from patients, adhering to protocols and guidelines approved by the Severance Hospital Institutional Review Board and Committee on Human Research (3–2020‐0006). Tissue samples were obtained from nine patients who underwent epilepsy surgery at Severance Hospital, Seoul, Korea (Table 2). Five patients were diagnosed with TLE associated with hippocampal sclerosis (HS), confirmed through preoperative MRI and postoperative histopathological evaluation by independent neuropathologists. Four patients, without evidence of neuronal loss or gliosis, but diagnosed with other epileptic syndromes (one with Lennox–Gastaut syndrome, one with West syndrome, and two with lateral temporal lobe epilepsy), were classified as the No HS group.

**TABLE 2:** Overview of the clinical cases.

Case No.	Gender	Age	Hippocampus pathology	Duration of epilepsy	CP	GTC	MRI
1	Male	16	Histologically normal	15y	+	+	N
2	Male	11	Histologically normal	10y	−	−	N
3	Female	4	Histologically normal	3.8y	−	−	N
4	Female	19	Histologically normal	7y	+	+	N
5	Male	56	HS type1	41y	−	+	MTS
6	Male	30	HS type1a	17y	+	+	MTS
7	Male	16	HS type 1b	3y	−	+	MTS
8	Female	44	HS type1b	37y	+	−	MTS
9	Female	30	HS type1	17y	+	−	MTS

Abbreviations: CP, complex partial seizure; GTC, generalized tonic–clonic seizure; HS, hippocampal sclerosis; MRI, magnetic resonance imaging; MTS, mesial temporal sclerosis; N, normal.

For surgical specimen blocks, the resected brain tissue was fixed in phosphate‐buffered 4% PFA overnight, cryoprotected in 30% sucrose solutions, frozen in OCT on dry ice, and stored at −80°C. Cryostat‐cut sections (20 μm thick) were collected and placed on glass slides. Paraffin‐embedded human hippocampal tissues were deparaffinized by passing them through a xylene and ethanol series, followed by citrate antigen retrieval as previously described (Zhu et al. 2024). After deparaffinization, the tissues were thoroughly washed in 0.01 M PBS containing 0.01% Triton X‐100 (PBST) and then incubated with a blocking solution of 3% bovine serum albumin in PBST for 1 h at room temperature. Subsequently, the sections were incubated overnight at 4°C with a mixture of primary antibodies (Table 1). The following day, the sections were rinsed and incubated with secondary antibodies (Table 1) for 2 h. Finally, the stained sections were covered by a glass coverslip using a hard‐set antifade mounting medium containing DAPI (Vectashield; Vector Laboratories). Images were captured using a confocal laser‐scanning microscope (LSM 700; Carl Zeiss).

To evaluate TRPV5 expression in human hippocampal tissue, we performed semiquantitative scoring of TRPV5 immunoreactivity within Iba1‐positive microglia, adapted from a previously described method (Jeong et al. 2018; Pirttila et al. 2005). Briefly, TRPV5 signal intensity in Iba1‐positive microglia was scored as follows: 0, no double‐positive cells; 1, few TRPV5‐positive microglia; 2, a moderate number of TRPV5‐positive microglia; 3, a high number of TRPV5‐positive microglia.

### 2.5 Stimulation of Primary Hippocampal Microglia and Treatment With a TRPV5 Inhibitor

Adult C57BL/6J mice were euthanized by cervical dislocation and decapitated. The brains were removed and placed in ice‐cold dissection media containing minimal essential medium supplemented with 1% penicillin–streptomycin, 2.5% HEPES buffer solution, and 1% 1 M Tris solution (all from Gibco, CA, USA). The hippocampi were subsequently isolated, dissociated, and cultured in complete media, including Dulbecco's modified Eagle's medium F12 supplemented with 10% fetal bovine serum, 1% penicillin–streptomycin, and 1% GlutaMAX (all from Gibco) for 6 h. Primary hippocampal microglia were isolated as previously described (Woolf et al. 2021). The isolated microglia were randomly allocated to different treatment groups and incubated in T25 flasks at 37°C in a humidified 5% CO_2_ atmosphere for 4 days. The cells were subsequently harvested and plated onto culture plates or cover glasses for each experiment (4 × 10^4^ cells/well). To evaluate microglial activation in response to lipopolysaccharide (LPS) from *Escherichia coli* (L2630, Sigma‐Aldrich), microglial cells were divided into three groups: (1) a control group with no exposure to LPS, (2) a group exposed to 10 ng/mL LPS, and (3) a group exposed to 100 ng/mL LPS for 24 h. To evaluate the effect of TRPV5 inhibition on LPS‐induced microglial activation, cultured microglial cells were treated with econazole (Eco; S5492; Selleck Chemicals, Munich, Germany), a potential TRPV5 inhibitor (De Jesus‐Perez et al. 2024; Hughes et al. 2018; Yan et al. 2011), prior to LPS treatment. Microglial cells were divided into three groups: (1) no econazole treatment, (2) group treated with 0.1 μmol/L econazole, and (3) group treated with 1.0 μmol/L econazole.

### 2.6 Immunocytochemistry

Primary cultured cells were washed with Dulbecco's phosphate‐buffered saline (dPBS; Gibco), fixed in 4% PFA/4% sucrose for 15 min at room temperature, and washed with dPBS. The cells were subsequently permeabilized with 0.01% Triton X‐100 in dPBS for 15 min. After blocking in 5% bovine serum albumin (Bovogen Biologicals, MEL, Australia) in dPBS for 1 h, the cells were incubated overnight at 4°C in dPBS containing primary antibodies (Table 1). This was followed by incubation with the appropriate fluorescence‐conjugated secondary antibodies (Table 1) for 2 h at room temperature. After washing, the cells were counterstained with Hoechst 33258 (Molecular Probes, Eugene, OR, USA) for 20 min at room temperature. The cells were then mounted on slides and examined under a fluorescence microscope (Axio Imager M2). Images were captured using a confocal laser‐scanning microscope (LSM 700).

### 2.7 Microglia Morphology Analysis and Quantification of TRPV5 Expression

Microglial cells were categorized based on their morphological characteristics, as previously described (He et al. 2021). Cells exhibiting long, thin processes with small somas were classified as resting microglia, whereas those exhibiting short, thick processes with enlarged somas were identified as activated microglia. Cell counting was performed manually based on these morphological features across at least five randomly selected fields per sample. The percentages of resting and activated microglia were calculated for each experimental condition.

We utilized corrected total cell fluorescence (CTCF) quantification to quantify the fluorescence intensity of TRPV5 in microglia cells exposed to varying concentrations of the inhibitor (0.1 and 1 μmol/L), along with LPS stimulation, as previously described (Yaqubi et al. 2023). In brief, CTCF values were calculated by manually delineating microglial cells to extract parameters such as average total microglial area, mean intensity, and integrated density. Adjacent background selections were subsequently performed within the same region of each microglial cell to provide a reliable measure for subsequent comparisons. These extracted values were then used to compute individual CTCF metrics, using the following CTCF equation:

$$\begin{array}{c} \text{CTCF}=\text{Integrated density}\;-\;\left(\text{area of selected cell}\right)\\ \;\times \;\text{mean fluorescence of background readings} \end{array}$$

### 2.8 Seizure Monitoring

To assess seizure onset parameters, mice were continuously monitored from the time of pilocarpine administration until termination of seizure activity. As previously described (Jeong et al. 2021; Park et al. 2024), the latency to the first convulsive seizure (corresponding to stage 3 on Racine's scale) was defined as the time elapsed until the onset of forelimb clonus, and the onset time of SE was determined by the appearance of sustained, generalized tonic–clonic seizures.

### 2.9 Effect of TRPV5 Suppression in Activated Microglia After Pilocarpine‐Induced SE in Vivo

To evaluate the effects of TRPV5 inhibition on SE‐induced microglial activation and inflammatory responses, mice were treated with a TRPV5 inhibitor (Eco; 40 mg/kg; i.p.) or vehicle for three consecutive days before pilocarpine treatment. Econazole was initially dissolved in 100% ethanol and diluted in a vehicle solution containing 5% Tween 80, 5% polyethylene glycol 400 (Sigma‐Aldrich), and 4% ethanol immediately before injection. A 1 h after seizure termination with diazepam, mice received their fourth injection of econazole or vehicle, followed by daily injections for three additional days. Mice were sacrificed 4 days after SE induction.

### 2.10 Image Analysis of Double Immunofluorescence Staining

In the experiment of double immunofluorescence staining, the immunoreactivity of TRPV5‐positive cells and Iba1‐expressing microglia was analyzed in the subpyramidal area of the hippocampus between bregma −1.46 and −2.30 mm, as previously described (Park et al. 2022; Zhu et al. 2024). Quantitative analyses were performed using Fiji software (ImageJ; National Institutes of Health, Bethesda, MD, USA).

### 2.11 Western Blot Analysis

Western blot samples were prepared as previously described (Jeong et al. 2024). In brief, isolated whole hippocampal tissue or cultured primary microglial cells were resuspended in sodium dodecyl sulfate (SDS) lysis buffer or radioimmunoprecipitation assay (RIPA) lysis buffer, respectively, and centrifuged at 4°C for 15 min at 14,000 × g. The protein concentration in the supernatant was quantified using a bicinchoninic acid assay kit (Thermo Scientific, Rockford, IL, USA). Proteins were separated using SDS‐polyacrylamide gel electrophoresis with appropriate molecular weight markers (Thermo Scientific) and transferred onto polyvinylidene difluoride membranes (Millipore) using an electrophoretic transfer system (Bio‐Rad Laboratories, Hercules, CA, USA). After blocking with 5% skim milk in 1X Tris‐buffered saline with 0.1% Tween 20 (TBST) for 1 h, the membranes were incubated overnight at 4°C with specific primary antibodies (Table 1). After washing, the membranes were incubated with secondary antibodies (Table 1), and the blots were developed using ECL western blotting detection reagents (Amersham Biosciences, Piscataway, NJ, USA). Protein bands were subsequently measured using the ImageQuant 800 computer imaging device (Amersham) and accompanying software.

### 2.12 Statistical Analysis

Data were analyzed using GraphPad Prism 9 software (GraphPad Software Inc., San Diego, CA, USA). All data are presented as the means ± standard error of the mean (SEM), with individual data points indicated. One‐way analysis of variance (ANOVA) followed by Tukey's post hoc test was used for multiple groups. Statistical significance was set at *p* < 0.05. The specific *p*‐values and sample sizes for each analysis are provided in the figure legends.

## 3 Results

### 3.1 Expressional Changes of TRPV5 in the Hippocampus After Pilocarpine‐Induced SE

**FIGURE 1:**
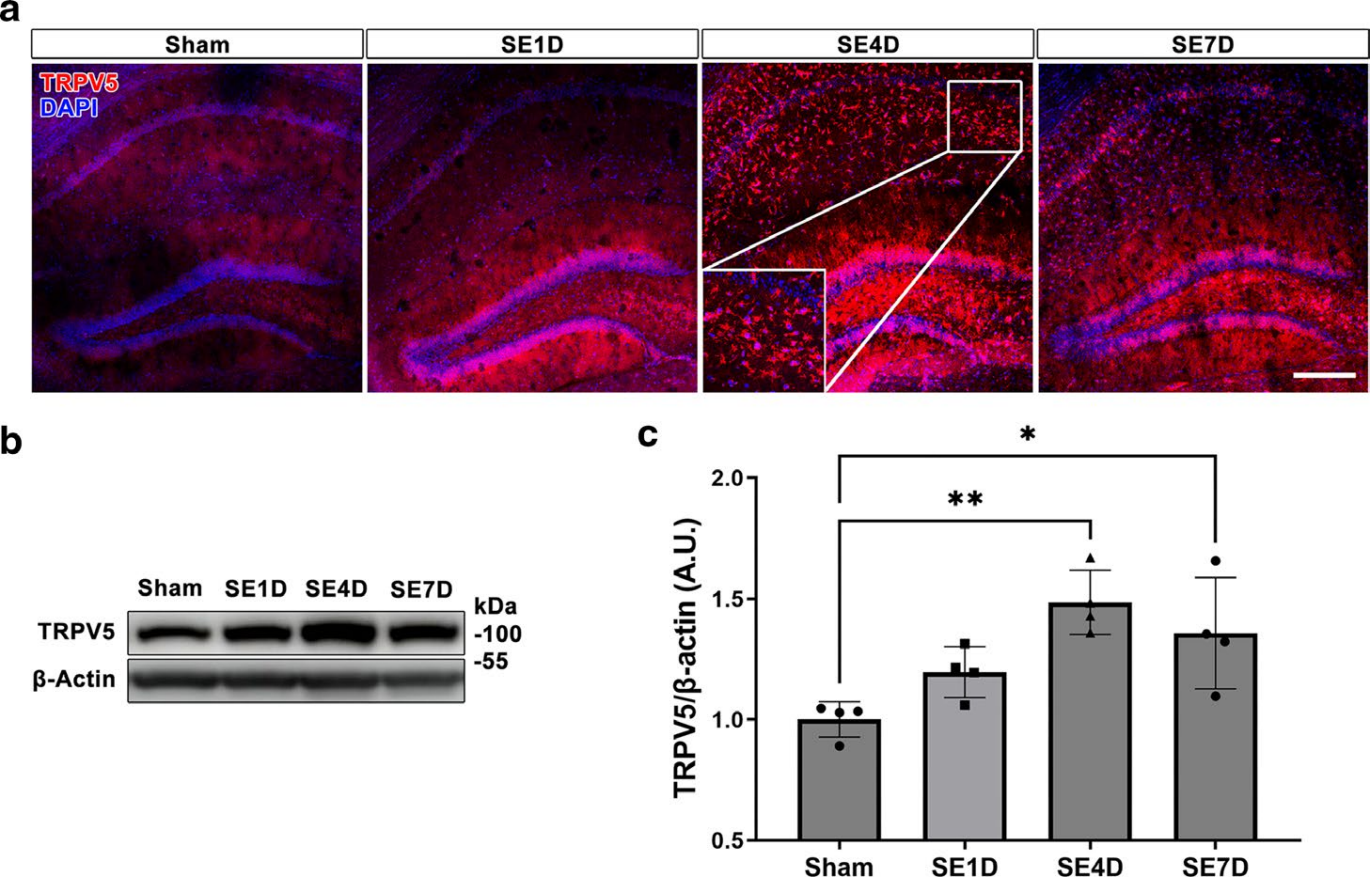
Temporal changes in TRPV5 expression after SE induction. (a) Representative TRPV5 immunohistochemistry images of hippocampal CA1 regions from sham and SE‐induced mice at various time points. Notably, glial‐like TRPV5 expression was markedly elevated 4 days after SE induction, gradually declining by 7 days post‐SE onset in the affected hippocampus. Scale bar = 200 μm. (b) Western blot analysis demonstrated a significant elevation in TRPV5 protein levels at 4 and 7 days post‐SE induction. (c) The histogram presents a quantitative analysis of TRPV5 protein band density, normalized to β‐Actin for each sample [ANOVA followed by Tukey's post hoc test, F (3,12) = 8.099, *p* = 0.0032, *n* = 4 for each group]. Values are presented as mean ± SEM. *p < 0.05, **p < 0.01; significantly different from Sham.

To investigate the changes in TRPV5 expression after SE induction, we initially examined its distribution and expression in the mouse hippocampus after SE onset. In the hippocampus of sham‐manipulated mice, TRPV5 was slightly expressed in the pyramidal neurons of the CA1 and CA3 areas, as well as in dentate granule neurons (Figure 1a). In the hippocampus lesioned by SE onset, glial‐like expression of TRPV5 was markedly elevated 4 days after SE induction, with expression gradually decreasing 7 days post‐SE onset (Figure 1a). Consistent with the immunostaining results, TRPV5 protein levels were significantly upregulated at 4 days (*p* = 0.003 vs. sham) and 7 days (*p* = 0.022 vs. sham) post‐SE onset compared with sham‐manipulated mice (Figure 1b,c).

### 3.2 Cellular Phenotype of TRPV5‐Positive Cells in the Hippocampus After SE

**FIGURE 2:**
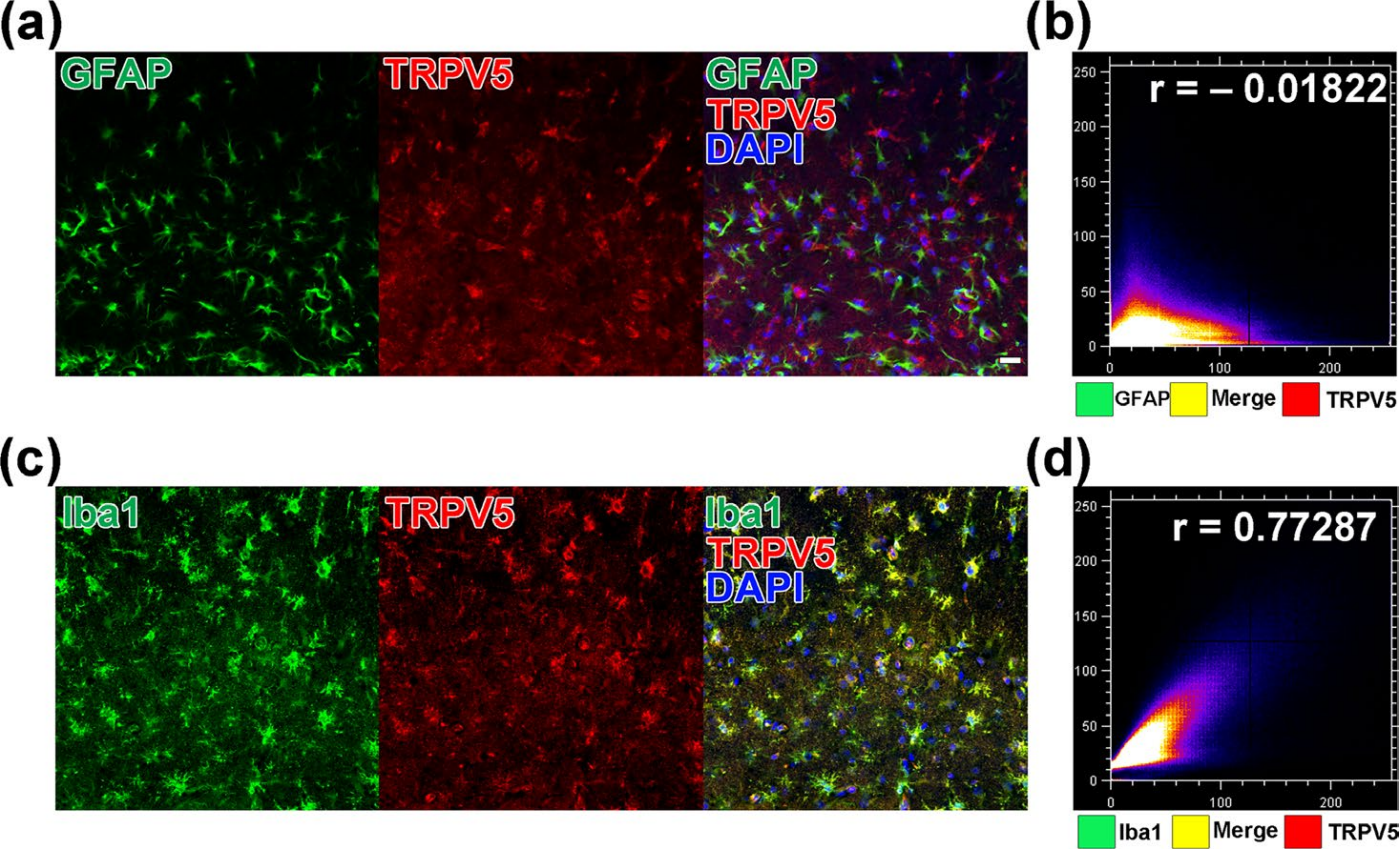
Cellular phenotype of TRPV5 expression in glial cells after SE onset. (a) Representative double immunohistochemical images of GFAP‐positive astrocytes (green) and TRPV5 (red) in the hippocampus 4 days after SE onset. Notably, upregulated TRPV5 was not observed in GFAP‐positive astrocytes, despite an increase in astrocyte presence within the hippocampus post‐SE. Scale bar = 20 μm. (b) Scatter plot showing the Pearson's correlation coefficient quantifying TRPV5 and GFAP colocalization (*r* = −0.01822 ± 0.03191, *n* = 4 for each group) in the hippocampus post‐SE. (c) Representative double immunohistochemical staining images for Iba1‐positive microglia (green) and TRPV5 (red) in the hippocampus 4 days post‐SE. TRPV5 expression is predominantly elevated in Iba1‐positive microglial cells. (d) Scatter plot depicting the Pearson's correlation coefficient for TRPV5 and Iba1 colocalization (*r* = 0.77287 ± 0.022141, *n* = 4 for each group) in the hippocampus following SE.

Given the upregulation of glial‐like TRPV5 expression in the hippocampus after SE induction, we performed double immunofluorescence staining with TRPV5 and either GFAP or Iba1, markers for astrocytes and microglia, respectively. Colocalization analysis was subsequently conducted to identify the cellular phenotypes of TRPV5‐positive cells. In the results of double immunofluorescence staining with TRPV5 and GFAP, the colocalization analysis revealed a weak correlation between TRPV5 expression and GFAP‐positive astrocytes (*r* = −0.01822 ± 0.03191; Figure 2a). Subsequent double staining with TRPV5 and Iba1 revealed a strong colocalization between TRPV5 expression and Iba1‐labeled microglia (*r* = 0.77287 ± 0.022141; Figure 2b), indicating a significant localization of TRPV5 expression within activated microglia. These findings indicate that TRPV5 is predominantly expressed in activated microglia following SE induction.

### 3.3 Increased TRPV5 Expression Within Activated Microglia in Epileptic Patients With Hippocampal Sclerosis

**FIGURE 3:**
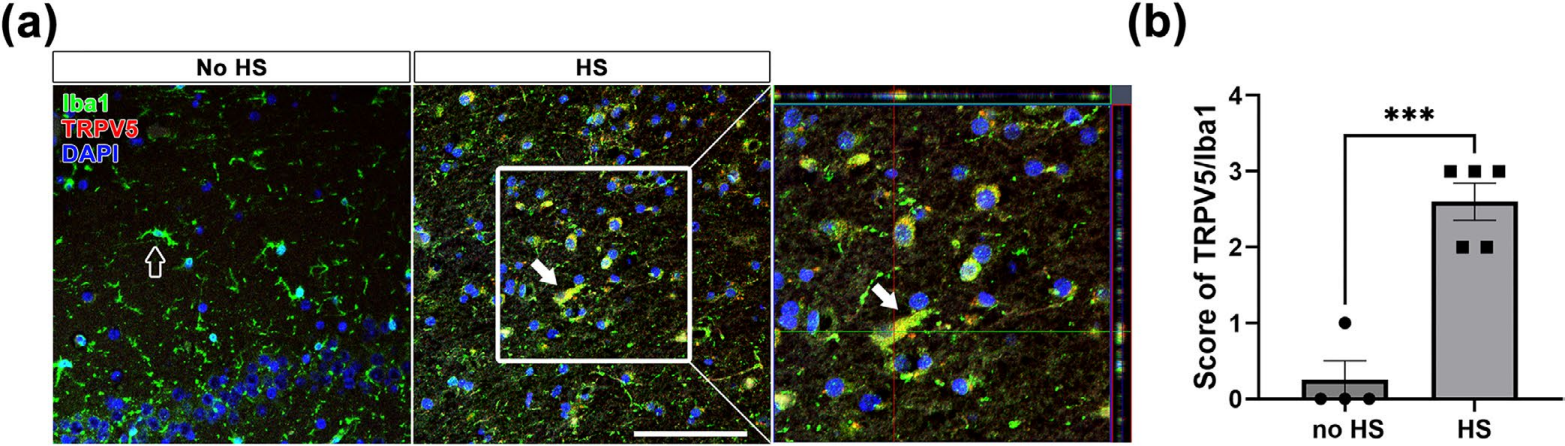
TRPV5 upregulation in activated microglia in the sclerotic hippocampal tissues of patients with temporal lobe epilepsy (TLE). (a) Representative immunostaining images of Iba1 (green) and TRPV5 (red) in non‐sclerotic control and sclerotic hippocampal tissues from TLE patients. Notably, TRPV5 expression is markedly enhanced in activated microglia within the HS group as indicated by the filled arrow. Scale bar = 100 μm. (b) Semiquantitative analysis of TRPV5 immunoreactivity within Iba1‐positive microglia shows a significant increase in the HS group compared to controls [Student's *t*‐test, t(7) = 6.637, *p* < 0.001, *n* = 4 for the No HS group, *n* = 5 for the HS group]. All values are presented as mean ± SEM with individual data points shown. ***p < 0.001; significantly different from the no‐HS group.

We examined whether TRPV5 expression was upregulated in activated microglia within hippocampal specimens from TLE patients with HS using double immunofluorescence staining for TRPV5 and Iba1. In histologically normal hippocampi (no HS), Iba1‐positive microglia appeared to be in a resting state and did not co‐localize with TRPV5 expression (Figure 3a, empty arrow). Conversely, TRPV5 expression was increased in activated microglia in the hippocampal tissues from TLE patients with HS (Figure 3a, filled arrow). These results indicate that TRPV5 is upregulated in activated microglia under epileptic conditions. Furthermore, semiquantitative analysis of TRPV5 immunoreactivity within Iba1‐positive microglia revealed a significant increase in TRPV5 expression in TLE patients with HS compared with the no HS group (Figure 3b; *p* = 0.004). These findings suggest that elevated TRPV5 expression could be associated with activated microglia during the epileptic process.

### 3.4 Effect of TRPV5 Inhibition on LPS‐Induced Activation of Primary Hippocampal Microglia

**FIGURE 4:**
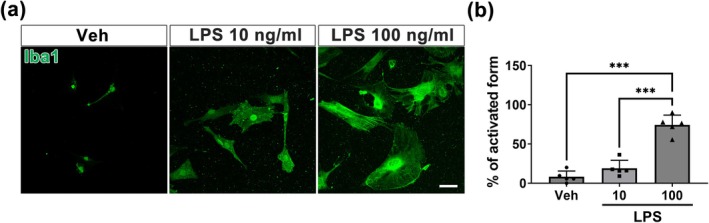
TRPV5 expression in LPS‐induced activated primary microglia. (a) Immunocytochemistry images showing TRPV5‐Iba1 double‐positive primary cultured microglia treated with varying concentrations of LPS (10 or 100 ng/mL) for 24 h. LPS treatment induces notable changes in microglial morphology. Scale bar = 20 μm. (b) Graphical representations of the percentages of microglia in activated states under each treatment condition. Note that the percentage of activated microglia significantly increased following LPS treatment [ANOVA followed by Tukey's post hoc test, F (2,12) = 59.95, *p* < 0.001, *n* = 5 for each group]. All values are presented as mean ± SEM with individual data points shown. ***p < 0.001; significantly different from Veh.

Generally, LPS treatment activates microglia to release proinflammatory cytokines, making it a widely used method for establishing neuroinflammatory models in vitro (Zhang et al. 2022). Based on this, we investigated whether TRPV5 is involved in the microglia‐mediated inflammatory response following LPS stimulation in vitro. To examine the activation of microglia by LPS treatment, we subsequently exposed microglia to various concentrations of LPS (10 and 100 ng/mL). Vehicle‐treated microglia exhibited a polarized morphology with thin processes, indicating a resting form (Figure 4a). In contrast, microglia treated with 10 ng/mL LPS showed altered morphology, characterized by shortened processes and enlarged cell bodies (Figure 4a). Microglia exposed to 100 ng/mL LPS displayed a more pronounced change in morphology, adopting an amoeboid‐like shape indicative of the activated microglial form (Figure 4a). Quantification of microglial morphologies under different conditions revealed a significant increase in activated microglia following LPS treatment compared to the vehicle‐treated group (*p* < 0.001 vs. Veh; Figure 4b). These results demonstrated the successful establishment of neuroinflammatory models in vitro. As the proportion of activated microglia was higher with the 100 ng/mL dose, this concentration was used for all subsequent experiments.

**FIGURE 5:**
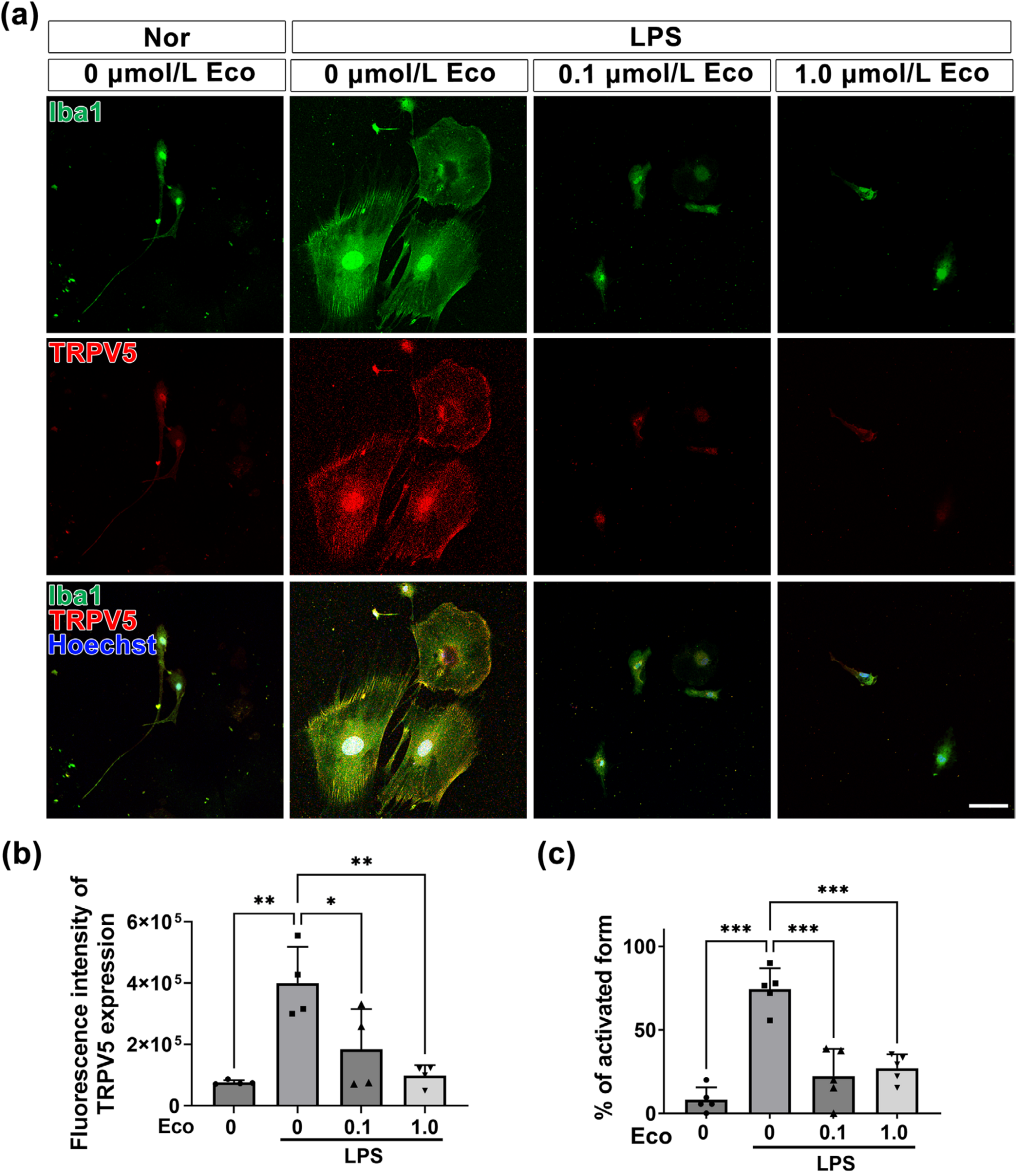
TRPV5 inhibition suppresses LPS‐induced microglia activation in vitro. (a) Immunocytochemistry images showing TRPV5‐Iba1 double‐positive cells in primary cultured microglia, treated with different doses of the TRPV5 inhibitor, econazole (0.1 or 1.0 μmol/L), for 24 h prior to LPS stimulation. Econazole treatment induced noticeable alterations in TRPV5 expression, microglial morphology, and activation status compared to untreated controls in LPS‐stimulated cells. Scale bar = 20 μm (b) Bar plot of the fluorescence intensity of TRPV5 expression under various conditions. Econazole treatment significantly reduced the elevated fluorescence intensity of TRPV5 within LPS‐activated microglia [ANOVA followed by Tukey's post hoc test, F (3,12) = 10.79, *p* = 0.001, *n* = 4 for each group]. (c) Graph showing the percentage of activated microglia under various conditions. Both doses of econazole treatment suppressed microglia activation in the LPS‐treated groups [ANOVA followed by Tukey's post hoc test, F (3,16) = 30.52, *p* < 0.001 *n* = 5 for each group]. All values are presented as mean ± SEM with individual data points shown. *p < 0.05, **p < 0.01; ***p < 0.001; significantly different from each group.

Next, to clarify the influence of TRPV5 on microglial activation, we used immunofluorescence staining to investigate whether TRPV5 suppression by econazole (Eco), a structural inhibitor of TRPV5 (De Jesus‐Perez et al. 2024; Hughes et al. 2018), could induce morphological changes in microglial cells following LPS stimulation. The results of immunofluorescence staining showed that LPS treatment induced microglial activation with increased TRPV5 expression; however, econazole treatment (0.1 and 1.0 μmol/L) suppressed both TRPV5 expression and LPS‐induced morphological changes in microglial cells (Figure 5a). Quantitative analysis further indicated that the increased fluorescence intensity of TRPV5 in LPS‐activated microglia was significantly reduced by econazole treatment (*p* = 0.024, LPS vs. LPS_Eco 0.1; *p* = 0.002, LPS vs. LPS_Eco 1.0; Figure 5b), suggesting that econazole is a potent inhibitor of TRPV5 (Yan et al. 2011). Additionally, quantitative analysis revealed that econazole treatment significantly attenuated LPS‐induced microglial activation, with a decrease in the percentage of activated microglia (*p* < 0.001, LPS vs. LPS_Eco 0.1; *p* < 0.001, LPS vs. LPS_Eco 1.0; Figure 5c). These results suggest that TRPV5 inhibition could regulate microglial activation.

### 3.5 TRPV5 Suppression Mitigated Inflammatory Responses During Microglial Activation Induced by LPS

**FIGURE 6:**
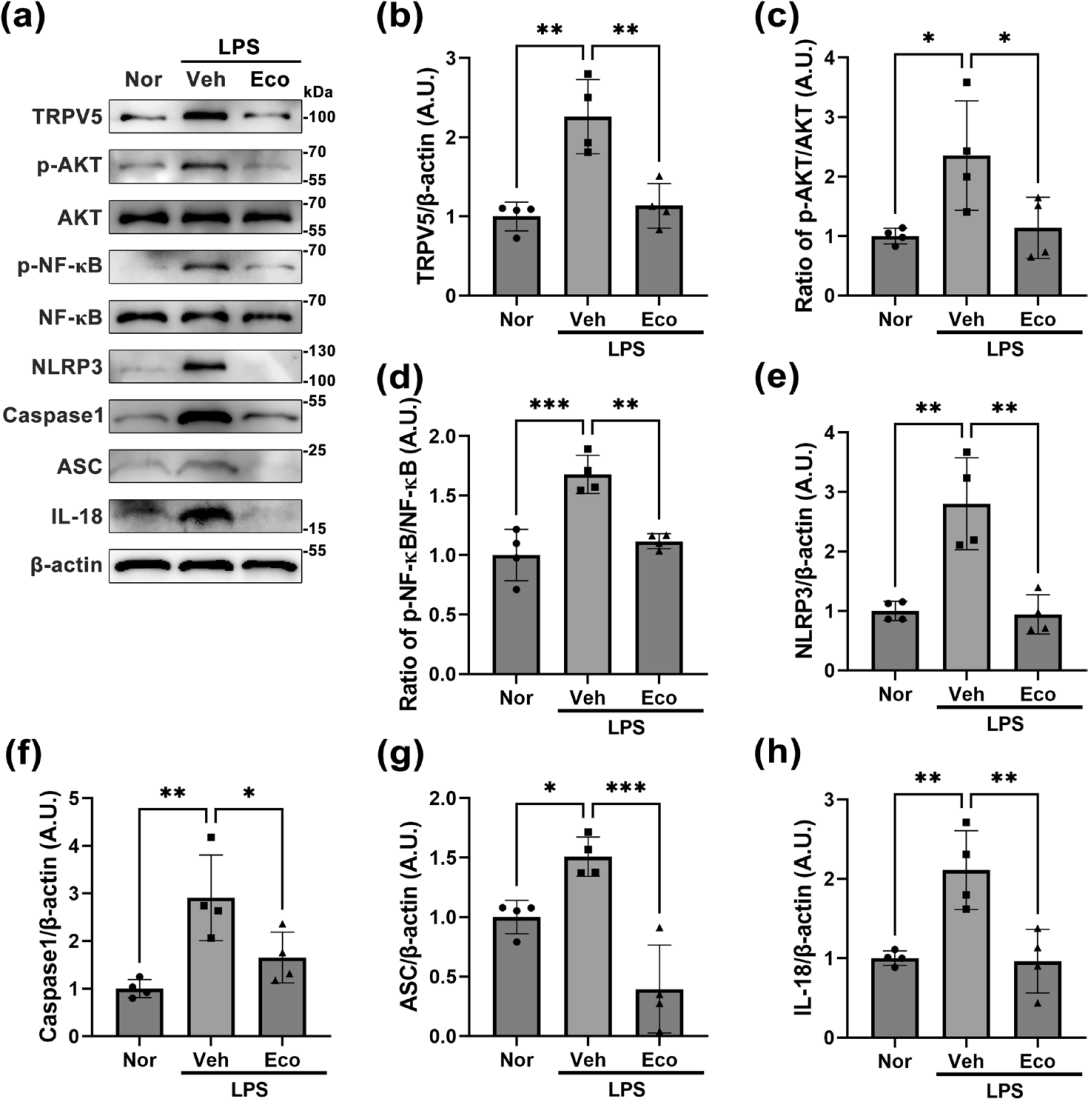
TRPV5 Inhibition alleviates the activation of AKT/NF‐κb pathway and the formation of the NLRP3 inflammasome complex in activated microglia. (a) Representative western blots showing the expression levels of TRPV5, p‐AKT, AKT, p‐NF‐κB, NF‐κB, NLRP3, Caspase1, ASC, IL‐18, and β‐Actin expression in each experimental group. Note that LPS treatment increased the levels of TRPV5, p‐AKT, AKT, p‐NF‐κB, NF‐κB, NLRP3, Caspase1, ASC, and IL‐18 in primary cultured microglia compared to normal microglia, while pharmacological inhibition of TRPV5 reduces these protein levels compared with the LPS group stimulated microglia. (b) Histogram depicting the quantitative analysis of TRPV5 protein bands normalized to β‐Actin expression [ANOVA followed by Tukey's post hoc test, F (2,9) = 9.664, *p* = 0.0057]. (c) Histogram showing the ratio of p‐AKT to total AKT protein bands normalized to β‐Actin expression [ANOVA followed by Tukey's post hoc test, F (2,9) = 5.880, *p* = 0.023]. (d) Histogram illustrating the ratio of p‐NF‐κB to NF‐κB protein bands normalized to β‐Actin expression [ANOVA followed by Tukey's post hoc test, F (2,9) = 20.68, *p* < 0.001]. **(e)** Histogram representing NLRP3 expression [ANOVA followed by Tukey's post hoc test, F (2,9) = 18.28, *p* < 0.001]. (f) Histogram showing Caspase1 expression [ANOVA followed by Tukey's post hoc test, F (2,9) = 9.936, *p* = 0.005]. (g) Histogram of ASC expression levels [ANOVA followed by Tukey's post hoc test, F (2,9) = 20.31, *p* < 0.001]. (h) Histogram showing IL‐18 expression [ANOVA followed by Tukey's post hoc test, F (2,9) = 12.30, *p* = 0.003]. All values are presented as the mean ± SEM with individual data points indicated (*n* = 4 for each group). *p < 0.05, **p < 0.01, ***p < 0.001; significantly different from each group.

Based on our results (Figure 5), we further investigated whether TRPV5 inhibition affects microglia‐derived inflammatory processes, using western blot analysis (Figure 6a). The higher dose of econazole treatment (1.0 μmol/L) resulted in a greater inhibition in TRPV5 expression within activated microglia (Figure 5b); therefore, the dose of econazole was standardized at 1.0 μmol/L for the experiment. Consistent with our results (Figure 5b), econazole treatment reduced the elevated TRPV5 protein level in LPS‐stimulated microglia (*p* = 0.001, Nor vs. LPS_Veh; *p* = 0.003, LPS_Veh vs. LPS_Eco; Figure 6b). Moreover, TRPV5 inhibition by econazole significantly suppressed the ratio of phosphorylated protein kinase B (AKT) to total AKT (*p* = 0.049, LPS_Veh vs. LPS_Eco; Figure 6c) and the ratio of phosphorylated nuclear factor‐κB (NF‐κB) to total NF‐κB (*p* = 0.002, LPS_Veh vs. LPS_Eco; Figure 6d). Additionally, TRPV5 inhibition by econazole significantly limited the production of the nucleotide‐binding and oligomerization domain‐like receptor family pyrin domain‐containing 3 (NLRP3) inflammasome complex in activated microglia, including NLRP3, apoptosis‐associated speck‐like protein containing a caspase‐recruitment domain (ASC), and Caspase1, compared to LPS‐activated microglia treated with vehicle (NLRP3: *p* = 0.001, LPS_Veh vs. LPS_Eco, Figure 6e; Caspase1: *p* = 0.043, LPS_Veh vs. LPS_Eco, Figure 6f; ASC: *p* < 0.001, LPS_Veh vs. LPS_Eco, Figure 6g). Moreover, TRPV5 suppression significantly reduced the protein levels of interleukin‐18 (IL‐18), a proinflammatory cytokine, compared to the LPS‐treated group (*p* = 0.005, LPS_Veh vs. LPS_Eco; Figure 6h). Overall, these results suggest that TRPV5 is a potential regulator of microglia‐derived inflammatory processes via regulating the AKT/NF‐κB/NLRP3 pathway.

### 3.6 Effect of TRPV5 Inhibition on Seizure Activity

**FIGURE 7:**
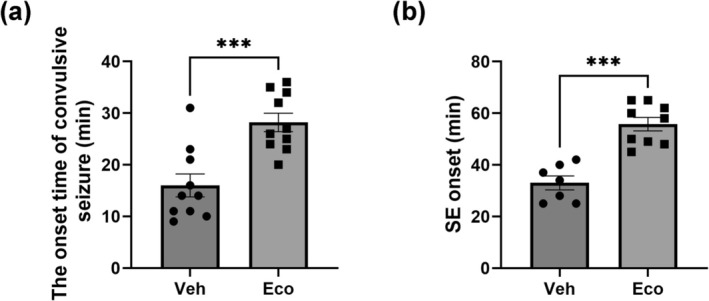
Effect of TRPV5 inhibition on seizure activity following pilocarpine‐induced status epilepticus (PCSE) in mice. (a) Pretreatment with Econazole significantly delayed the onset time of convulsive seizure (i.e., stage 3) in mice after pilocarpine administration compared to the vehicle‐pretreated group [Student's *t*‐test, t(18) = 4.297, *p* < 0.001, *n* = 10 per group]. (b) The onset time of SE (i.e., latency of SE onset) was also significantly increased in Econazole‐pretreated mice relative to the vehicle‐pretreated group [Student's *t*‐test, *t* (14) = 6.026, *p* < 0.001, *n* = 7 for Veh and *n* = 9 for Eco]. All values are presented as the mean ± SEM with individual data points shown. ***p < 0.001; significantly different from the vehicle group.

To evaluate the effect of TRPV5 inhibition on pilocarpine‐induced seizure activity, we analyzed the onset time of convulsive seizures and latency to SE induction in vivo. Econazole pre‐treatment significantly delayed both the onset of convulsive seizures and the latency to SE compared to the vehicle‐treated SE group (Figure 7a,b; onset time of convulsive seizures: *p* < 0.01 vs. Veh; latency to SE onset: *p* < 0.01 vs. Veh). These findings suggest that TRPV5 activity may contribute to seizure susceptibility during epileptogenesis.

### 3.7 TRPV5 Inhibition Restrained Microglial Activation and Inflammatory Processes in a Mouse Model of PCSE

**FIGURE 8:**
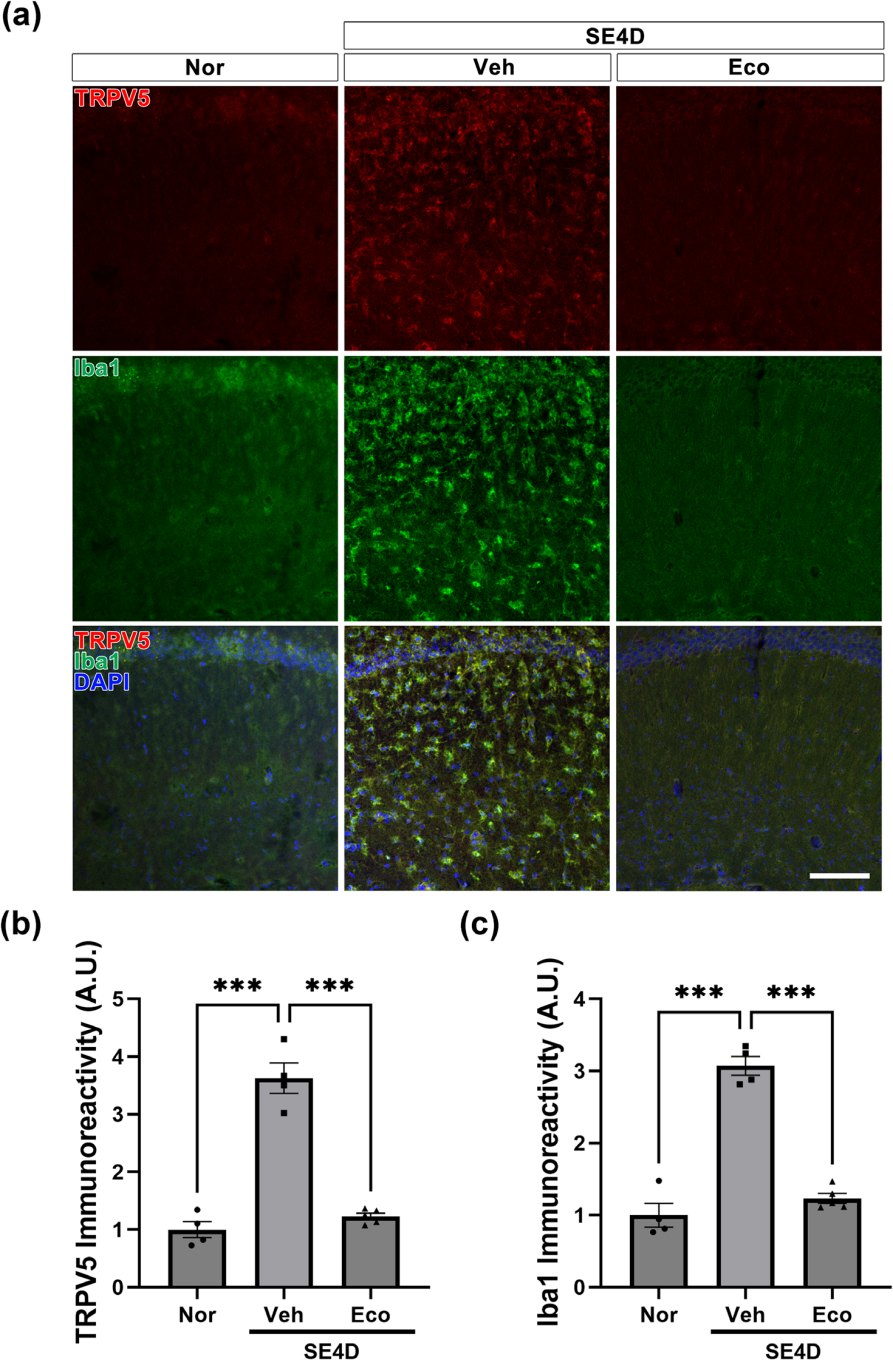
TRPV5 inhibition attenuates SE‐induced microglial activation in vivo. (a) Representative double immunohistochemistry images showing that TRPV5 upregulation (red) within activated microglia (green) 4 days after SE is decreased in the econazole‐treated group. Scale bar = 100 μm (b) Quantitative analysis of TRPV5 immunoreactivity expressed as the mean ± SEM with individual data points indicated [ANOVA followed by Tukey's post hoc test, F (2,10) = 76.57, *p* < 0.001]. (c) Quantitative analysis of Iba1 immunoreactivity presented as the mean ± SEM, with individual data points indicated [ANOVA followed by Tukey's post hoc test, F (2,10) = 85.31]. *n* = 4 for Nor and PCSE_Veh, *n* = 5 for PCSE_Eco. ***p < 0.001; significantly different from each group.

To examine whether our in vitro results were consistent with the effect of TRPV5 suppression in the lesioned hippocampus following epileptic seizures, we investigated the effect of TRPV5 inhibition by econazole on microglial activation and inflammatory processes in PCSE. Consistent with our in vitro results (Figure 5), the upregulation of TRPV5 immunoreactivity by SE induction was found to be significantly reduced by econazole treatment in the hippocampus (*p* < 0.001 vs. PCSE_Veh, *p* < 0.001 vs. PCSE_Eco; Figures 8a,b), indicating that econazole treatment acts as an effective TRPV5 inhibitor. Furthermore, TRPV5 suppression by econazole significantly alleviated SE‐induced microglial activation compared to the vehicle‐treated group (*p* < 0.001 vs. PCSE_Veh, *p* < 0.001 vs. PCSE_Eco; Figure 8a,c). These findings suggest that TRPV5 is involved in microglial activation in the SE‐lesioned hippocampus.

**FIGURE 9:**
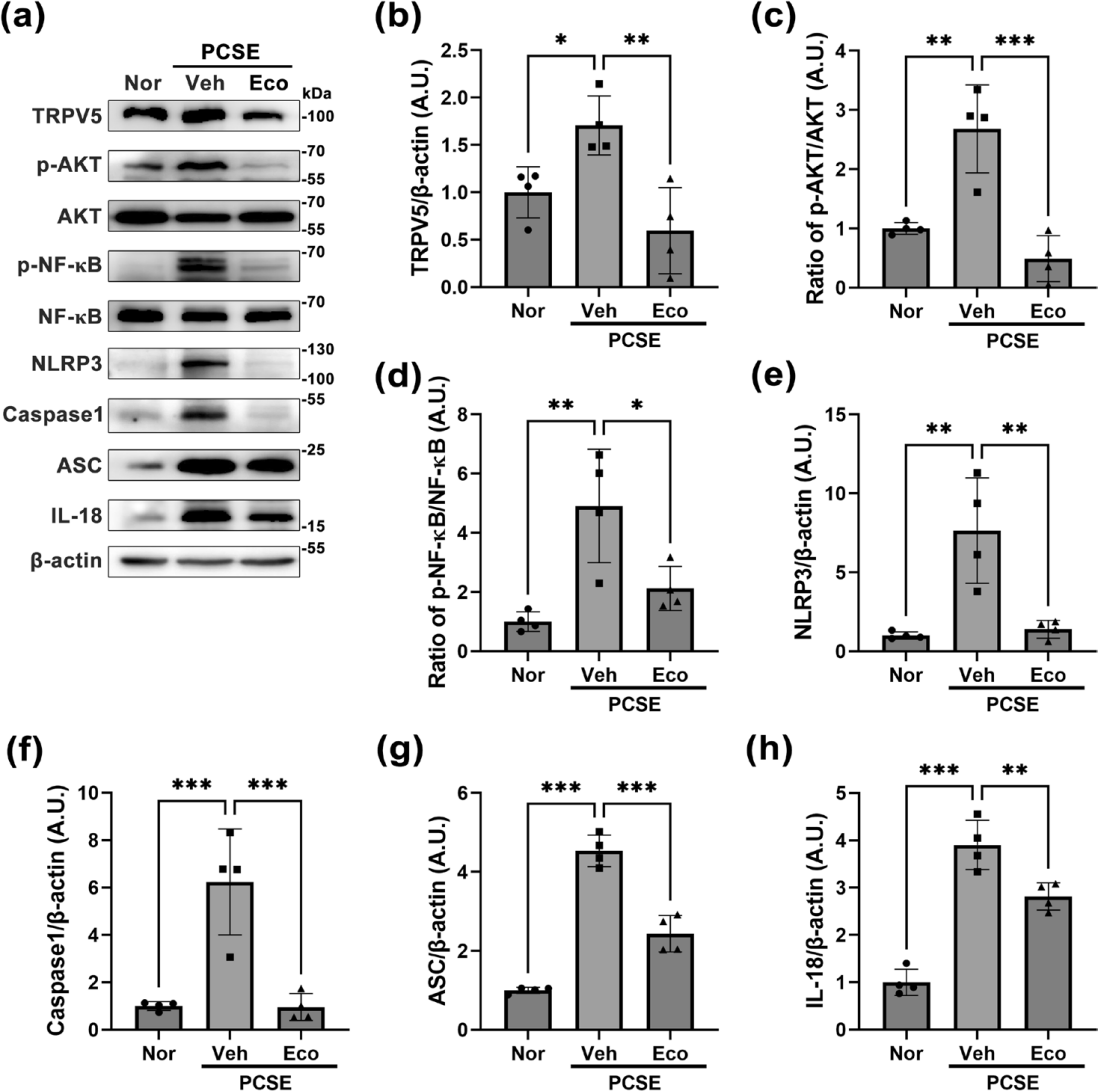
TRPV5 Inhibition reduces the production of NLRP3 inflammasome pathway by inhibiting the AKT/NF‐κB pathway in pilocarpine‐induced SE. (a) Representative western blots showing the expression of TRPV5, p‐AKT, AKT, p‐NF‐κB, NF‐κB, NLRP3, ASC, Caspase1, IL‐18, and β‐Actin across different experimental groups. Note that SE induction enhances the protein levels of TRPV5, p‐AKT, p‐NF‐κB, NLRP3, ASC, Caspase1, and IL‐18 in the hippocampus compared to the normal group. TRPV5 inhibition suppresses the elevations of these proteins induced by SE. (b) Histogram representing quantitative analysis of TRPV5 protein bands normalized to β‐Actin expression [ANOVA followed by Tukey's post hoc test, F (2,9) = 10.13, *p* = 0.005]. (c) Histogram depicting the ratio of phosphorylated AKT to total AKT (p‐AKT/AKT), normalized to β‐Actin expression [ANOVA followed by Tukey's post hoc test, F (2,9) = 22.05, *p* < 0.001]. (d) Histogram showing the ratio of phosphorylated NF‐κB to total NF‐κB (p‐NF‐κB/NF‐κB), normalized to β‐Actin expression [ANOVA followed by Tukey's post hoc test, F (2,9) = 11.19, *p* = 0.004]. (e) Histogram data showing the protein levels of NLRP3, normalized to β‐Actin [ANOVA followed by Tukey's post hoc test, F (2,9) = 14.47, *p* = 0.002]. (f) Quantification of Caspase1 expression, normalized to β‐Actin [ANOVA followed by Tukey's post hoc test, F(2,9) = 20.58, *p* < 0.001]. (g) Histogram showing ASC expression, normalized to β‐Actin [ANOVA followed by Tukey's post hoc test, F (2,9) = 99.39, *p* < 0.001]. (h) Quantification of IL‐18 expression, normalized to β‐Actin [ANOVA followed by Tukey's post hoc test, F (2,9) = 59.79, *p* < 0.001]. All values are presented as the mean ± SEM with individual data points indicated (*n* = 4 for each group). *p < 0.05, **p < 0.01, ***p < 0.001; significantly different from each group.

We further investigated the anti‐inflammatory effects of TRPV5 inhibition on PCSE using western blot analysis (Figure 9A). Consistent with our in vitro results (Figure 6), econazole treatment inhibited the significant increase in TRPV5 protein level in SE‐induced hippocampus (*p* = 0.004, PCSE_Veh vs. PCSE_Eco; Figures 9a,b). Moreover, our results showed that TRPV5 inhibition by econazole treatment induced a significant decrease in the ratio of phosphorylated AKT to total (*p* < 0.001, PCSE_Veh vs. PCSE_Eco; Figure 9c) and the ratio of phosphorylated NF‐κB to total NF‐κB (*p* = 0.024, PCSE_Veh vs. PCSE_Eco; Figure 9d). Econazole treatment also suppressed the activation of the NLRP3 inflammasome, as evidenced by reduced protein levels of NLRP3(*p* = 0.004, PCSE_Veh vs. PCSE_Eco; Figure 9e), Caspase1 (*p* < 0.001, PCSE_Veh vs. PCSE_Eco; Figure 9f), and ASC (*p* < 0.001, PCSE_Veh vs. PCSE_Eco; Figure 9g). Furthermore, econazole treatment decreased the production of the proinflammatory cytokine IL‐18 (*p* = 0.007, PCSE_Veh vs. PCSE_Eco; Figure 9h). Overall, these results suggest that TRPV5 is involved in microglia‐associated neuroinflammation through activating the AKT/NF‐κB/NLRP3 pathway in epileptic brain injury.

## 4 Discussion

Previous studies have shown that TRPV channels, including TRPV1 and TRPV4, are involved in epileptogenic processes, demonstrating their roles in the pathological mechanisms of epilepsy. However, prior to this study, the involvement of TRPV5 in neuropathological conditions, particularly epilepsy, remained unknown. The present study investigated the role of TRPV5 in microglial activation following epileptic seizures, noting significant upregulation of TRPV5 in activated hippocampal microglia in both experimental models and patients with TLE. Pharmacological inhibition of TRPV5 effectively suppressed microglial activation and neuroinflammatory responses by modulating AKT/NF‐κB signaling and NLRP3 inflammasome‐mediated proinflammatory cytokine production in both in vitro and in vivo models. To the best of our knowledge, these findings represent the first evidence indicating a novel role of TRPV5 in microglial activation and associated neuroinflammation following epileptic seizures.

Microglia, which are resident macrophages in the CNS, play critical roles in immune surveillance and homeostasis maintenance by constantly monitoring the brain parenchyma (Bernier et al. 2020). Nevertheless, pathological insults, such as epileptic seizures, induce a reactive state in microglia, characterized by morphological changes—including enlarged cell bodies and shortened, thickened processes—as well as the release of proinflammatory mediators, such as cytokines and chemokines (Avignone et al. 2015; Yu et al. 2023). This reactive transformation contributes to the neuroinflammatory response and exacerbates disease progression. In line with this evidence, previous studies have demonstrated that pharmacological inhibition of microglial activation using minocycline (Moller et al. 2016; Tikka et al. 2001) reduces neuroinflammatory responses, attenuates seizure severity, and mitigates neuronal damage in both kainic acid and PCSE models (Abraham et al. 2012; Wang et al. 2015). These findings indicate that activated microglia are crucial pathological manifestations following epileptic seizures. In the present study, our results revealed a distinct spatial pattern of TRPV5 expression, specifically showing the predominant upregulation of TRPV5 in activated microglia in the hippocampus of PCSE mice and patients with TLE. This upregulation was also observed in LPS‐stimulated primary microglial cells, while pharmacological inhibition of TRPV5 with econazole mitigated microglial activation. Therefore, our observations suggest that TRPV5 is involved in microglial activation under pathological conditions, such as epileptic insults.

Studies have reported that activated microglia not only release proinflammatory mediators but also engage in multiple intracellular signaling pathways that amplify the neuroinflammatory response (Rodriguez‐Gomez et al. 2020). Among these, AKT signaling is a vital intracellular pathway that drives proinflammatory responses in LPS‐stimulated BV2 microglial cells (Cianciulli et al. 2016). Phosphorylated AKT functions as an upstream regulator of NF‐κB activation, a key transcription factor involved in the production of inflammatory cytokines (Guo et al. 2024). This pathway is integral to the priming and activation of the NLRP3 inflammasome, a mediator of neuroinflammation (Bauernfeind et al. 2009; Guo et al. 2015). Based on these facts, our results revealed that TRPV5 inhibition suppresses AKT phosphorylation, reducing NF‐κB activation in both in vitro and in vivo models. This disruption of upstream signaling cascades contributes to impaired activation of the NLRP3 inflammasome, as evidenced by the observed reductions in inflammasome components and IL‐18 production. Our findings further show that TRPV5 is involved in the modulation of microglial activation and microglia‐mediated neuroinflammation following an epileptic insult.

Econazole is an imidazole antifungal drug used to treat superficial candidiasis, dermatophytosis, pityriasis versicolor, and skin contagions (Srivastava et al. 2021). Prior studies have shown that econazole possesses anticancer activity in a broad range of cancers. For instance, econazole promotes apoptosis in Adriamycin‐resistant breast cancer cells by suppressing the phosphoinositide 3 kinase/AKT pathway (Zhang et al. 2022). Interestingly, several studies have further identified econazole as an effective pharmacological inhibitor of TRPV5. Structural studies using cryo‐EM demonstrated that econazole directly inhibits TRPV5 activity by targeting the pore region of its channels (De Jesus‐Perez et al. 2024; Hughes et al. 2018). Furthermore, econazole suppressed the protein level of TRPV5 in osteoclast‐like cells (Yan et al. 2011). This suggests that econazole is a potent inhibitor of TRPV5. In the present study, econazole effectively reduced TRPV5 expression in activated microglia following epileptic seizure and LPS stimulation, and further induced the disruption of AKT/NF‐κB activation, NLRP3 inflammasome complex assembly, and the production of proinflammatory cytokine IL‐18. The attenuation of neuroinflammation observed in both in vitro and in vivo models underscores econazole as a TRPV5 inhibitor and provides a foundation for future therapeutic exploration.

Overall, the present study indicates a potential role for TRPV5 in microglia‐mediated neuroinflammation following epileptic seizures. Although our findings demonstrated the ability of TRPV5 inhibition to attenuate neuroinflammatory pathways, the involvement of TRPV5 in other pathogenic mechanisms underlying neurological disorders currently remains unclear. Prior research has reported TRPV5 upregulation in a rat osteoarthritis model, in which TRPV5 induces chondrocyte apoptosis via the mitogen‐activated protein kinase cascade and the AKT/mechanistic target of the rapamycin pathway in osteoarthritis (Wei et al. 2017). Together with our observations, this evidence led us to conclude that TRPV5 may serve as a potential regulator of several cellular and molecular mechanisms, such as microglial activation and excessive inflammatory action, implicated in the pathogenesis of various neurological disorders, including epilepsy. Further exploration of the functional spectrum of TRPV5 will provide valuable information regarding its therapeutic potential in diverse neuropathological conditions.

**FIGURE 10:**
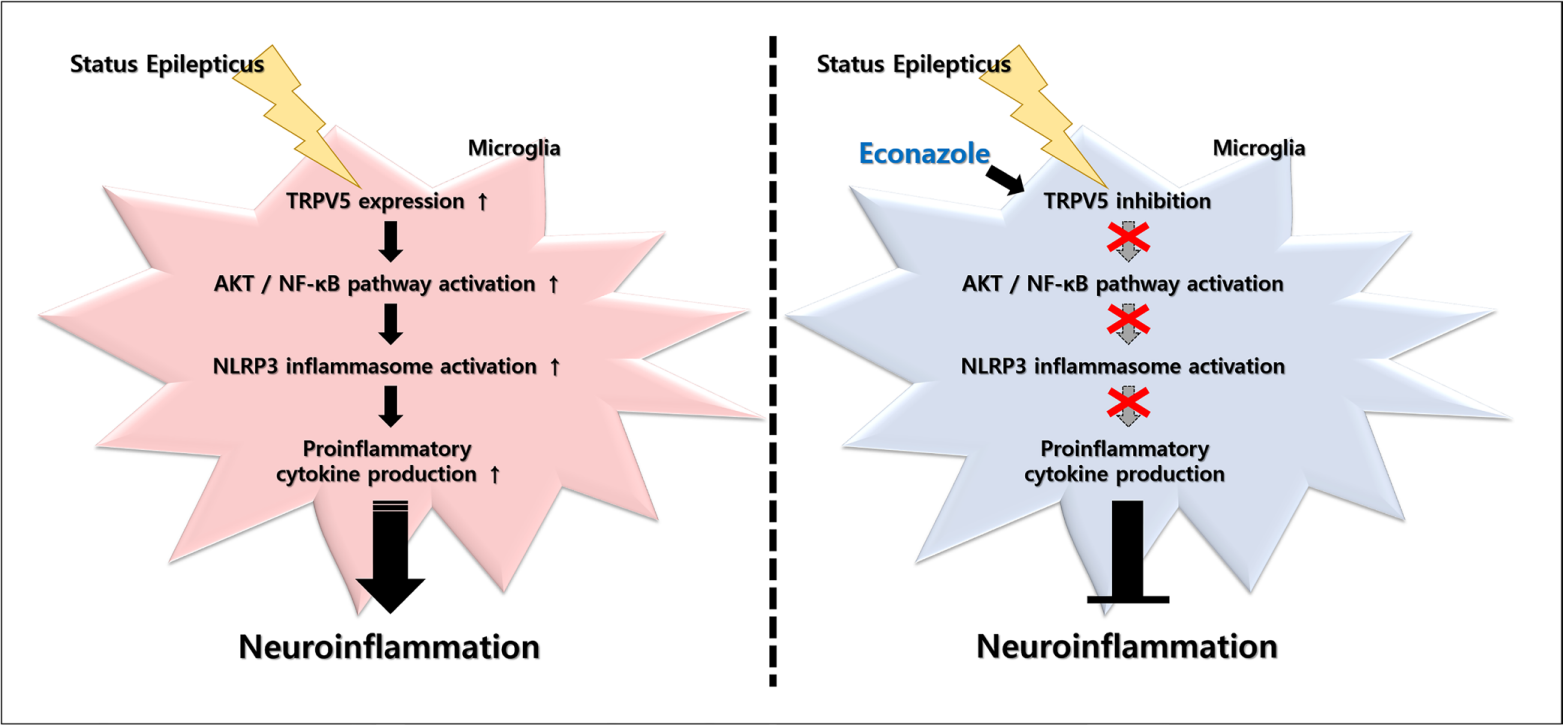
Schematic representation showing the proposed role of TRPV5 in microglial activation‐mediated neuroinflammation following epileptic seizures. The present study suggests that TRPV5 is upregulated in activated microglia following prolonged seizure activity, such as that induced by SE. TRPV5 upregulation in activated microglia is involved in neuroinflammation via activating AKT/NF‐κB/NLRP3 pathway, resulting in proinflammatory cytokine production. These cascades are reversed by treatment with Econazole, a potent TRPV5 inhibitor. Taken together, these findings support a potential crucial role for TRPV5 inhibition in controlling excessive neuroinflammatory process in the hippocampus during the sub‐acute phase of epilepsy. Taken together, these findings highlight TRPV5 inhibition as a promising strategy to control excessive neuroinflammatory processes in the hippocampus during the sub‐acute phase of epilepsy.

In conclusion, the present study demonstrates a molecular mechanism illustrating the potential role of TRPV5 in microglia‐mediated neuroinflammation during sub‐acute seizures (Figure 10). Our results showed the upregulated expression of TRPV5 in activated hippocampal microglia in both experimental models and patients with TLE. Furthermore, pharmacological blockade of TRPV5 using econazole was found to regulate several inflammatory processes via suppressing AKT/NF‐κB signaling, NLRP3 inflammasome activation, and proinflammatory cytokine production in microglia activation induced by epileptic seizures. Taken together, these findings suggest that TRPV5 is involved in microglial activation and microglia‐mediated neuroinflammatory processes following SE. This study provides new insights into TRPV5 as a potential therapeutic target for mitigating neuroinflammation during epileptic progression.

## Author Contributions

**Soojin Park:** investigation, validation, analysis, writing ‑ original draft, preparation. **Se Hoon Kim:** contribution of human tissue. **Chul Hoon Kim:** contribution of the project administration. **Kyoung Hoon Jeong:** conceptualization, funding acquisition, writing ‑ review and editing, supervision. **Won‐Joo Kim:** writing ‑ review and editing, supervision.

## Ethics Statement

Ethical approval and consent to participate in tissue experiments were conducted with informed consent from patients, adhering to the protocols and guidelines approved by the Severance Hospital Institutional Review Board and Committee on Human Research (3–2020‐0006). All procedures were approved by the Institutional Committee for the Care and Use of Laboratory Animals at Yonsei University Health System (IACUC 2023–0208).

## Consent

All authors have reviewed the final manuscript and consent to publication.

## Conflicts of Interest

The authors declare no conflicts of interest.

## Data Availability

The data that support the findings of this study are available from the corresponding author upon reasonable request.
